# Cardiological aspects of Fabry disease: from diagnosis to therapeutic efficacy assessment

**DOI:** 10.1186/s13023-026-04485-1

**Published:** 2026-07-09

**Authors:** Francesca Graziani, Elena Biagini, Alessia Argirò, Rodolfo Citro, Guido Iaccarino, Chiara Lanzillo, Ines Monte, Federico Pieruzzi, Cinzia Zuchi, Maurizio Pieroni

**Affiliations:** 1 Department of Cardiovascular Sciences-CUORE, Fondazione Policlinico Universitario A. Gemelli IRCCS, Largo A. Gemelli 8, 00168 Rome, Italy; 2 Cardiology Unit, Cardiac Thoracic and Vascular Department, IRCCS University Hospital of Bologna, Bologna, Italy; 3 European Reference Network for Rare, Low Prevalence and Complex Diseases of The Heart-ERN GUARD-Heart, Bologna, Italy; 4 Department of Experimental and Clinical Medicine, University of Florence, Florence, Italy; 5 Department of Medicine and Health Science, University of Molise, Campobasso, Italy; 6 Department of Vascular Pathophysiology, IRCCS Neuromed, Pozzilli, Isernia, Italy; 7 Department of Clinical Medicine and Surgery, Federico II University, Naples, Italy; 8 U.O.C. Cardiology Unit, Santo Spirito in Sassia Hospital, Rome, Italy; 9 CHIRMED Department, University of Catania, Catania, Italy; 10 Nephrology, Fondazione IRCCS San Gerardo dei Tintori, Monza, Italy; 11 School of Medicine and Surgery, University of Milano-Bicocca, Milan, Italy; 12 Division of Cardiology and Cardiovascular Physiopathology, Hospital and University of Perugia, Perugia, Italy; 13 Cardiomyopathy Unit, Careggi University Hospital, Florence, Italy

**Keywords:** Biomarkers, Cardiovascular disease, Cardiovascular magnetic resonance, Echocardiography, Fabry disease, Metabolic disorders

## Abstract

**Background:**

Fabry disease (FD) is a rare inborn error of metabolism in which absent or deficient activity of α-galactosidase A leads to lysosomal dysfunction and globotriaosylceramide accumulation in different organs, including the heart. FD is characterized by a broad clinical spectrum, but Fabry cardiomyopathy represents the leading cause of morbidity and mortality. Electrocardiogram (ECG), echocardiography, and cardiac magnetic resonance (CMR) imaging are the cornerstones to suspect and diagnose cardiac involvement and to monitor it. Cardiac biomarkers such as troponin and N-terminal pro-B-type natriuretic peptide are also used to ascertain the degree of cardiac involvement. However, definitive data are lacking on how to plan the follow-up of patients with or without cardiac involvement, when to initiate specific therapy, and when to switch from one treatment to another. Current proposals suggest initiating treatment when there is evidence of left ventricular hypertrophy but before fibrosis develops.

**Main body:**

Ten experts on FD met in Milan on March 8, 2024. The panel of experts was selected based on their expertise and contribution to the literature on FD. The group deliberated on appropriate parameters for cardiac assessment at diagnosis and during follow-up. The panel discussed developing a multiparametric cardiac assessment system (including ECG, laboratory biomarkers, echocardiographic and CMR parameters) to be used in clinical practice to guide management and monitor treatment response. The authors defined the requirements of such a system and advocate for the research needed to build and validate it. The experts also proposed a multiparametric clinical pathway for cardiac assessment, treatment initiation, and follow-up, designed to support routine clinical decisions. This was intended as general guidance, acknowledging that clinicians should tailor management to the individual patient’s specific characteristics - a principle of particular importance in rare diseases such as FD, where organ involvement and treatment response can differ substantially among individuals.

**Conclusion:**

Since cardiovascular disease is a leading cause of death in FD, an agreed staging system for evaluating cardiac progression is urgently needed. Further research should be carried out to confirm the criteria of a cardiac assessment system, evaluate its prognostic and predictive validity, and ensure its reliability and reproducibility.

**Supplementary Information:**

The online version contains supplementary material available at https://doi.org/10.1186/s13023-026-04485-1.

## Background

Fabry disease (FD) is a rare X-linked inborn error of metabolism in which there is absent or deficient activity of a key lysosomal enzyme, α-galactosidase A (α-Gal A) [1]. The enzyme deficiency leads to intracellular accumulation of globotriaosylceramide (Gb3) in a range of cells, mostly vascular endothelial cells, renal cells, cardiac myocytes, and neurons [1]. FD is a multisystem condition in which affected individuals develop a range of signs and symptoms, including hearing loss, tinnitus, fatigue, paresthesia, skin rashes, nausea, vomiting, bloating, and corneal opacities, as well as progressive renal and cardiovascular dysfunction [1]. Based on residual α-Gal A activity, it is possible to categorize three distinct phenotypes of FD: (1) classical; (2) late onset; and (3) benign [2, 3]. The distinction into these three clinical phenotypes is easier in male than female patients, due to random X chromosome inactivation and its impact on residual α-Gal A activity in different organs in females [3]. Patients with FD have a reduced life expectancy compared with the general population, and cardiovascular disease is the leading cause of death [4].

In the last 20 years, different treatment options have become available for FD, including enzyme replacement therapy (ERT) and chaperone therapy (e.g. migalastat). Targeted treatments for FD have significantly improved quality of life and are proven to delay disease progression [5]. Existing guidelines emphasize the need for early introduction of ERT prior to the development of irreversible organ damage [6, 7]. In both the Canadian and European Fabry Working Group guidelines, patients with renal (elevated creatinine, proteinuria), cardiac (left ventricular [LV] mass, septal thickness, arrhythmia), neurologic (stroke, hearing loss, pain) or gastrointestinal involvement qualify for initiation of ERT [6, 7]. The European guidelines are less conservative, also advising initiating ERT in all male patients older than 16 years with classical FD prior to the development of symptoms or organ involvement [6, 7]. Furthermore, new forms of ERT, mRNA therapeutics, substrate reduction therapy and gene therapy are emerging [5].

Monitoring the progression of FD and treatment effects remains of paramount importance in guiding decision-making. Although cardiac imaging studies have provided important insights into cardiac disease progression at a tissue level [8–10], criteria for the staging of FD-related cardiac damage are lacking. Development of a common staging system for FD is complicated by the rarity of the disease (which hinders the possibility of performing adequately sized prospective studies), genotypic and phenotypic heterogeneity, and gender differences related to the X-linked nature of the disease [11].

A meeting of Italian FD experts was held with the aim of reviewing appropriate parameters for monitoring cardiovascular disease progression in patients with FD. This article summarizes the recommendations that emerged during the discussion and the unanswered questions/future research priorities.

## Methods

A meeting of 10 FD experts (the authors of this review) was held in Milan on March 8, 2024. The meeting was sponsored by Sanofi, who selected the participants based on their expertise in FD and contribution to the literature on the topic.

The authors identified four overarching themes: (i) early diagnosis of FD and baseline assessment of cardiac damage; (ii) monitoring of cardiac damage for disease staging; (iii) cardiac parameters to guide FD treatment decisions; and (iv) unmet needs in cardiac involvement of FD. The meeting was recorded and summarized, and the discussion was used as a framework for this review, supported by relevant published literature identified by the authors.

### Spectrum and pathogenesis of cardiac involvement in FD

Because of its rarity and the multiplicity of presenting signs, there are often considerable delays between the onset of symptoms and the diagnosis of FD. In the International Fabry Outcomes Survey (FOS), the median time between symptom onset and diagnosis was 4 years in children and 10.5 years in adults [12].

Cardiac involvement in FD is usually not observed early in life; myocyte accumulation of Gb3 may trigger inflammation and cardiac hypertrophy, and eventually the development of fibrosis [10]. Cardiac involvement is present in about 39% of patients with FD at diagnosis [13]. Left ventricular hypertrophy (LVH) is a characteristic sign, present in 33% of untreated women and 53% of untreated men, and its prevalence increases with age [13]. Cardiac involvement with LVH is the most common cardiac manifestation of FD and is an important determinant of clinical outcomes [14]. In a contemporary study of 560 patients treated with ERT, 55% of patients had LVH at baseline. LVH has been found to correlate with α-Gal A activity levels; as such male patients more frequently have LVH than female patients [15]. LVH develops early in the course of classic FD; in a longitudinal study of 76 patients, LVH was present in 11.1% of those between the age of 20–29 years, 40.6% between 30 and 39 years, and 76.5% between 50 and 59 years [14]. Male patients experienced a greater increase in left ventricular mass index (LVMI) over time compared with female patients [14]. Maurizi and colleagues found that LVMI progression occurred mostly in men (17 of 39 [43%]) versus women (5 of 21 [24%]; *p* < 0.01); LVMI progression most frequently occurred in patients aged > 30 years (17 of 22 [77%]) [16]. The presence of LVH at the start of ERT was associated with LVMI progression (odds ratio 1.3, 95% confidence interval [CI] 1.1 to 2.6; *p* = 0.02).

The pathogenesis of cardiac involvement in FD includes several mechanisms [17, 18]. The accumulation of Gb3 in myocytes, vascular endothelial cells and smooth muscle cells, as well as in endocardial, conduction, and valvular tissue, triggers functional impairment in cardiac myocytes, and activates hypertrophic pathways. Gb3 accumulation also affects ion channel expression and the electrical properties of cardiac myocytes [17]. In addition, Gb3 and globotriaosylsphingosine (lyso-Gb3) may act as antigens, triggering a chronic inflammatory response and a myocarditis-like phenotype. Immune-mediated myocarditis is detectable upon histology in a significant proportion of patients with FD, and correlates with disease severity [19].

There is also growing evidence that dysfunctional lysosomes negatively affect mitochondrial function [20, 21]. In skeletal muscle cells, mitochondrial dysfunction leads to metabolic derangement and the Warburg effect (an overuse of aerobic glycolysis); as a result, patients with FD are easily fatigued and have poor exercise tolerance [20]. Alterations in energy metabolism occurred in the cardiac myocytes of a mouse model of FD, before the development of LVH, suggesting a causal role [22]. Similar alterations were observed in human fibroblasts [20].

Eventually, patients with FD develop cardiac complications, such as arrhythmias, heart failure (HF), and ischemic chest pain [13, 23–25]. The average age for the development of cardiac symptoms in untreated patients with FD is 31 years in men and 40 years in women [13].

### Cardiac assessment in the diagnosis and follow-up of FD

**Fig. 1:**
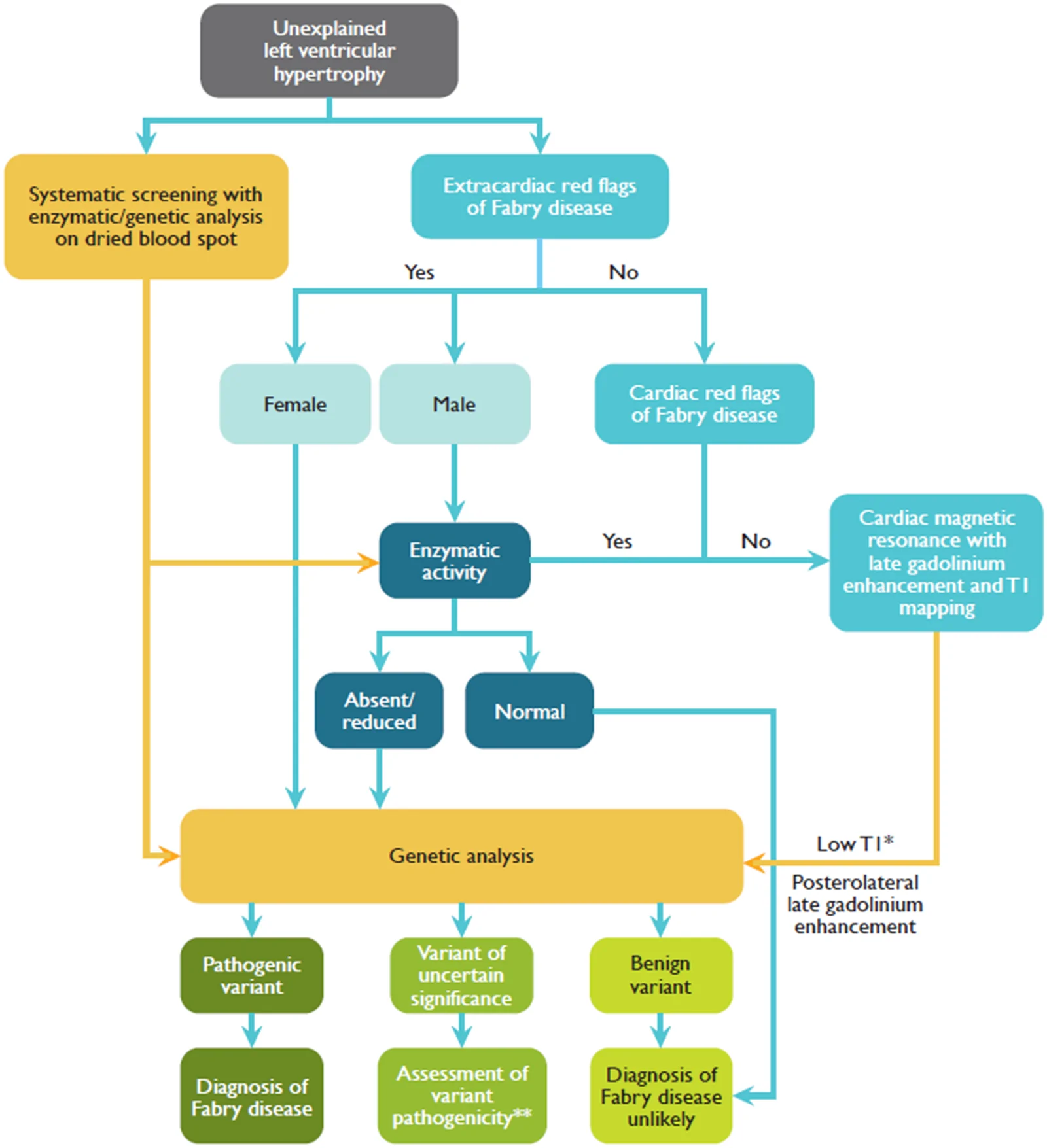
Proposed algorithm for the diagnosis of Fabry disease (FD) in patients with unexplained left ventricular hypertrophy (LVH) [17]. *low native T1 values reinforce or generate suspicion of FD; normal native T1 values do not exclude FD, being rarely observed in untreated patients with mild LVH (mostly female patients), or in advanced disease due to “pseudo-normalization”. With normal native T1 values, genetic analysis remains indicated if other findings suggest FD. **using lyso-Gb3 level assessment and endomyocardial biopsy. Lyso-Gb3, globotriaosylsphingosine. Originally published by and used with permission from the J*ournal of the American College of Cardiology*

Early diagnosis of FD and initiation of treatment is essential for limiting disease progression and delaying the onset of irreversible end-organ damage [26, 27]. Patients with undiagnosed FD may be referred for cardiac assessment if they have unexplained LVH, in which case clinical suspicion of FD may be aroused by the presence of extracardiac signs/symptoms, or the presence of particular ‘red flags’ on electrocardiography (ECG), echocardiography, and cardiovascular magnetic resonance (CMR) imaging (Table 1) [17, 28–34]. Fig. 1 outlines the steps in cardiac assessment and diagnosis of such patients [17].

**Table 1:** Diagnostic ‘red flags’ indicative of the presence of Fabry disease using cardiac assessment techniques [17, 28–32]

ECG	Echocardiography	Cardiovascular magnetic resonance
• Bradycardia • Reduced PR interval • ECG signs of LVH • Increased QRS duration • Conduction abnormalities (e.g. RBBB)	• LVH (usually symmetrical and concentric) with preserved LVEF • Binary sign (hyperechogenic endocardial surface adjacent to a hypoechogenic subendocardial layer) • Reduced global longitudinal strain (often affecting the inferolateral wall of the LV), global circumferential strain and RV longitudinal strain • Hypertrophy of papillary muscles • Mild-to-moderate aortic root dilation • Mitral and aortic valve thickening with mild-to-moderate regurgitation • LV diastolic dysfunction (increased E/e’ ratio, LA dilation) • RV hypertrophy	• Low native T1 times • Hypertrophy of papillary muscles • Basal inferolateral LGE • Elevation of T2 times in the inferolateral wall or in areas of LGE

E/e’, peak early mitral inflow velocity (E) divided by the early diastolic mitral annular velocity (e’); ECG, electrocardiography; LA, left atrium; LGE, late gadolinium enhancement; LV, left ventricle; LVEF, left ventricular ejection fraction; LVH, left ventricular hypertrophy; RBBB, right bundle branch block; RV, right ventricle

In addition to the early diagnosis of FD, cardiac parameters may also be used for monitoring progression and predicting future events.

### ECG

ECG is a powerful diagnostic tool for cardiomyopathies in general and for the detection of early cardiac involvement and the progression of FD. Key ECG features suggestive of FD are signs of LVH and conduction abnormalities [31, 33, 34].

In the early stages of FD, the PR interval is short [35]; a PR interval of ≤ 140 msec together with right bundle branch block (RBBB) were more likely associated with FD compared with sarcomeric hypertrophic cardiomyopathy [36]. Although PR intervals may remain within reference values, the PR interval and QRS duration are typically prolonged with progression of the disease [31, 35, 37]. The overall evolution of the ECG with the progression of FD exhibits a short PR interval at first, which then normalizes, and thereafter becomes longer than normal, with the appearance of bundle branch blocks [38]. The appearance of conduction disorders such as PR or QRS prolongation can be associated with fibrosis and can predict the onset of bradyarrhythmias [38].

ECG abnormalities may be evident before the development of LVH, and ECG characteristics may help in the differential diagnosis of the hypertrophic phenotype (Supplementary Table S1) [33, 34, 37, 39–41]. Parameters reported in more than one study include short PR interval [33, 36], increased P wave duration [34, 36], prolonged QRS duration [33, 40, 41], RBBB [33, 34, 36], and positivity of the Sokolow Lyon index [39, 40]. Several groups have developed scoring systems using ECG parameters that can help to identify patients with FD with reasonable accuracy [33, 37, 40].

A longitudinal study in 133 patients with FD showed that many ECG parameters change at a faster rate in those with FD than in unaffected individuals, including P wave duration, QRS duration, corrected QT intervals, Cornell index, spatial QRS-T angle and frontal QRS axis [35]. The rate of change was age- and sex-dependent, but the authors suggested that monitoring the rate of change would be a valuable way to monitor the development and progression of cardiac involvement [35].

Late complications of FD include the development of AV blocks and the consequent need for pacemaker implantation, atrial fibrillation (AF) and ventricular arrhythmias [42].

### Echocardiography

Echocardiography is usually the first imaging modality adopted in all patients with cardiomyopathies, due to its wide availability and low cost, and the same applies to FD [43]. The most frequent abnormalities described by standard transthoracic echocardiography are concentric LVH and papillary muscle thickening. Although strain analysis can unveil subtle abnormalities, ejection fraction is usually preserved, even in patients with no LVH [44]. On the other hand, diastolic function is often abnormal [28], but a restrictive pattern is seldom seen. Indeed, a recent study showed a more impaired diastolic function in patients with cardiac amyloidosis versus those with FD matched for LV wall thickness [45]. Moreover, the right ventricle (RV) is often hypertrophic with preserved RV systolic function [46] although strain analysis can reveal early RV impairment [43, 47]. Also, right atrial strain is impaired compared with age- and sex-matched healthy controls, even before LVH ensues [48].

Several studies have shown that echocardiographic parameters of cardiac damage (e.g., progression of LVH, left atrial dilation, worsening of diastolic dysfunction, development of reduced ejection fraction) are predictive of adverse cardiovascular outcomes in patients with FD. For example, in a study by Chang and colleagues, the severity of LVH (using the LVMI and global longitudinal strain [GLS]) predicted the 5-year occurrence of a composite outcome of all-cause mortality, HF hospitalizations, sustained ventricular tachycardia, and acute ischemic stroke [49]. A separate study found that the severity of LVH (by maximal wall thickness [MWT] or LVMI) and mean basal longitudinal strain were significant risk factors for de novo AF or cerebrovascular events [50], and correlates with the presence of ventricular arrhythmias [51]. Speckle tracking echocardiography-derived parameters can be useful as new prognostic markers in hypertrophic cardiomyopathies [51]. Moreover, LV-GLS on 3D echocardiography correlated with HF severity and HF outcomes (decompensation or cardiovascular hospitalization) [52]. These data indicate that echocardiography can be used reliably to follow-up patients with FD.

### CMR

CMR is an irreplaceable imaging technique to evaluate cardiomyopathies. CMR provides the differential diagnosis among the various causes of LVH through an accurate assessment of morphological, functional, and tissue characteristics [53, 54]. Moreover, compared with echocardiography, CMR has better reproducibility and is less likely to result in overestimates of LV mass or wall thickness [55].

Low native T1 values indicate sphingolipid deposition [28] and correlate with the histological severity of myocyte damage [56]. About 60% of patients with FD have low native T1 times before they have evidence of LVH [57]. The combination of low T1 and evidence of inflammation on the CMR in the presence of LVH is highly indicative of FD [53].

As FD progresses, patients may also show late gadolinium enhancement (LGE), a sign of myocardial fibrosis, at first in the inferolateral basal wall of the left ventricle and more extended in the late stages of the disease [58]. A multicenter study using CMR found that the pattern of gadolinium enhancement differed between patients with FD and those with chronic myocardial infarction (MI) or hypertrophic cardiomyopathy (HCM) [9]. Patients with FD with LGE had higher global and regional T2 values, particularly in the basal inferolateral region, than those without LGE. When compared with HCM or chronic MI in patients with LGE and with healthy individuals, those with FD and LGE also had higher T2 values in both septal/remote areas (where gadolinium enhancement would not be expected in the early phases of FD) and the basal inferolateral region [9].

As FD progresses, CMR features evolve. Low T1 values may persist, but T2 mapping becomes important once patients develop LGE. In patients with FD, T1 values are initially low, then they normalize, and eventually increase as cardiac damage worsens (due to the development of fibrosis), whereas T2 values increase progressively with worsening disease.

Augusto and colleagues found that when LGE is present, T2 is elevated in the LGE regions (usually in the basal inferolateral wall), with higher T2 values in patients with FD than in those with sarcomeric hypertrophy [9]. The T2 value was correlated with increased levels of troponin or N-terminal pro-B-type natriuretic peptide (NT-proBNP), as well as ECG and echocardiographic changes [9]. In a separate study, T2 was correlated with histological evidence of inflammatory infiltrates and troponin levels [59], supporting its role as a marker of cardiac damage. In the study by Augusto and colleagues, the basal inferolateral T2 value at baseline was a significant predictor of clinical worsening of FD; in contrast, baseline levels of troponin or NT-proBNP were not [9]. The presence of LGE, regardless of its location and extension, constituted a strong prognostic factor, conveying a more than three times higher risk of composite adverse outcomes and a more than four times higher risk of adverse cardiovascular outcomes [9]. Importantly, although LGE is usually a feature of advanced cardiomyopathy in FD, women may present with LGE before LVH development [60]. Orsborne and colleagues developed a model that predicts the 5-year risk of adverse cardiac outcome accurately for men and women with FD; this tool could easily be integrated into clinical care [61].

### Biomarkers

The plasma level of lyso-Gb3 is the key biomarker used to screen patients for FD [62], with levels above 2.0 ng/mL indicative of FD [63]. Lyso-Gb3 concentration is markedly elevated in adults with the classic FD phenotype; however, it is more variable in infants and adults with the late-onset phenotype and in women, some of whom have normal lyso-Gb3 levels despite confirmed disease [64]. For this reason, in these subgroups, clinicians should not rely on a single absolute measurement when interpreting borderline results, but instead perform serial monitoring of the trend over time.

Cardiac-specific biomarkers that may be used to identify cardiovascular involvement in FD include 3-nitrotyrosine (3-NT; a marker for endothelial nitric oxide dysfunction and vascular involvement) [62], cardiac troponin [65, 66], and NT-proBNP [67].

Markers of inflammatory activity (such as tumor necrosis factor [TNF], TNF receptor 1 and 2, galectin 1 and 3, and matrix metalloproteinases) are also elevated in patients with FD, and correlate with disease severity evaluated by CMR, renal function biomarkers and Mainz Severity Score Index (MSSI) [68]. Patients with FD exhibit a specific profile of small, noncoding mRNA (miRNA) suggestive of altered energy metabolism that is also consistent with reduced exercise tolerance and the presence of fatigue [21].

### Cardiopulmonary exercise testing

Patients with FD can safely undergo exercise testing [69], although they may present with a rapid onset of fatigue; indeed, exercise intolerance is common in FD [70]. Cardiopulmonary exercise testing has unique advantages and can quantify functional impairment, identify organ involvement, inform prognosis, assess therapeutic responses and uncover subclinical changes [70].

Exercise stress testing is included as an option for cardiac monitoring of patients with FD in the Chinese guidelines on the management of cardiac manifestations of FD [29]. Upon exercise testing, patients with FD show reduced exercise time [20, 71], lower functional capacity (maximum oxygen consumption [VO_2max_]) [39, 70], increased lactate production [20], slower heart rate at maximal exercise [71] and an increase in the ventilation/carbon dioxide production (VE/VCO_2_) slope (indicating reduced ventilatory efficiency) [50] compared with healthy individuals.

A correlation between exercise testing and CMR findings has been described previously. VO_2max_ indexed to body surface area is significantly associated with right ventricular volumes [71], and the VE/VCO_2_ slope is significantly associated with LV wall thickness on CMR, which is prognostic of the development of *de novo* AF or cerebrovascular events [50].

Using a Bruce protocol for exercise testing, it is possible to observe the prevalent anaerobic glycolytic metabolism, which is associated with a shorter than expected exercise tolerance [20]. It has been proposed that exercise tolerance might help to monitor response to therapy in patients with FD [20].

### Other assessments of cardiac function

Patients with FD typically have lower blood pressure (BP) than the general population [72]. However, hypertension can develop as FD progresses. The prevalence of hypertension in the Fabry population increases with age. Although the pathophysiology of hypertension in FD remains complex and is yet to be fully clarified, it can be a disease modifier. The key impact of concern is the effect of hypertension on cardio-renal dysfunction, specifically the progression to chronic kidney disease [72].

Thus, the monitoring of BP is advisable in patients with FD. BP monitors such as a 24-hour ambulatory BP monitor are useful devices that measure and record BP readings at 15–30-minute intervals over a 24-hour period, which provides a more accurate reflection of BP than a single reading [73].

### Cardiac staging in FD

**Fig. 2:**
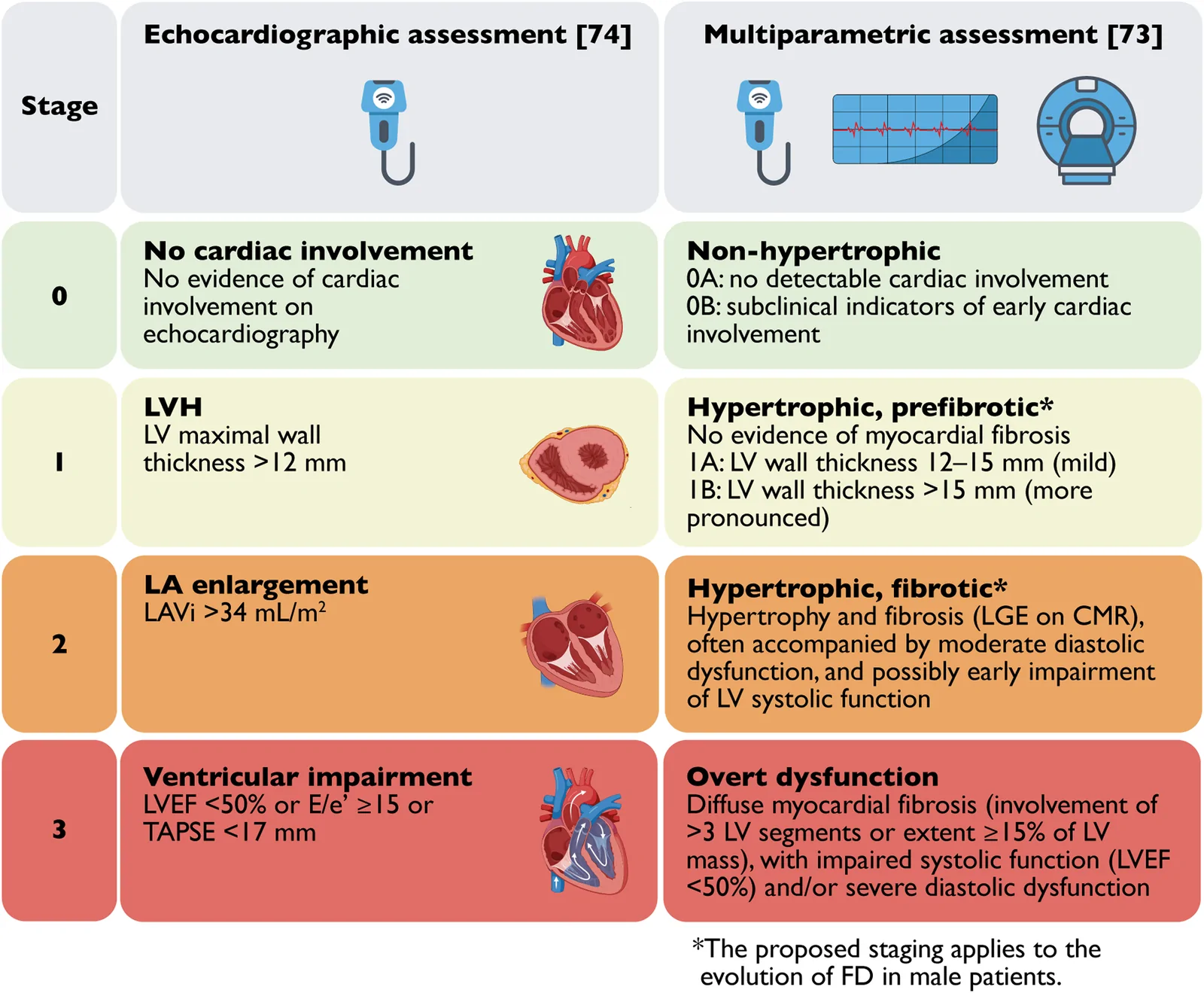
Two staging systems suggested for Fabry disease (FD) cardiac involvement [74, 75]. Schematic distribution of the features of the 4 disease stages proposed for FD. Part of the figure was created in BioRender. CMR, cardiac magnetic resonance; LAVi, left atrial volume indexed to body surface area; LGE late gadolinium enhancement; LV, left ventricle; LVEF, left ventricular ejection fraction; LVH, left ventricular hypertrophy; TAPSE, tricuspid annual plane systolic excursion

Two groups of investigators have suggested staging systems for cardiac involvement in FD (Fig. 2) [74, 75]. The first system proposed by Meucci and colleagues is based on echocardiographic parameters and identified four stages of FD: 0 – early (non-hypertrophic); 1 – hypertrophic; 2 – defined by the presence of LA enlargement; and 3 – functional impairment [75].

In a retrospective analysis in 314 patients with FD, the risk of an adverse cardiovascular outcome (composite of all-cause death, HF hospitalization, new-onset AF, major brady- or tachyarrhythmias or ischemic stroke) was significantly associated with the stage of cardiac involvement, with increasing risk for each stage increase (2% in stage 0; 16% in stage 1; 43% in stage 2; 69% in stage 3) [75]. Notably, cardiac damage staging provided a better risk stratification than the degree of LVH, even when adjusted for consolidated variables associated with disease prognosis [75].

The second system, proposed by Del Franco, also defines four stages: 0 – early (non-hypertrophic); 1 – hypertrophic pre-fibrotic; 2 – hypertrophic-fibrotic; and 3 – overt dysfunction [74]. This staging system describes a range of ECG, biochemical, echocardiographic and CMR parameters that define each stage [74]. The proposed staging applies to the evolution of FD in male patients, where fibrosis follows hypertrophy; this is less applicable to women, who can develop LGE before LVH [10].

While neither system has been investigated prospectively and proven to be associated with adverse outcomes, the staging system proposed by Meucci and colleagues has some advantages since it requires only one form of assessment (echocardiography) and has shown potential in predicting future events, albeit retrospectively [75]. When the population was re-staged after a median follow-up of 5 years, 25% of the study participants had stage worsening. Predictors of stage worsening were age, male sex, classic phenotype, and renal failure [76].

The stages described by Del Franco and colleagues [74] may be a useful starting point, but it has not been validated clinically and any system should include clearly defined thresholds for parameters in order to be reliable and reproducible.

As well as containing clinically relevant parameters, the staging system may need to include weighting, similar to that in the FAbry STabilization indEX (FASTEX) [77]. FASTEX includes three cardiac parameters (LVH, ECG parameters/arrhythmias, and the New York Heart Association [NYHA] functional class), two renal function parameters and two neurological parameters, and correlates well with other clinical assessment tools (Fabry Disease Severity Scoring System [DS3] and MSSI) [77]. A multiparametric cardiac assessment system could improve on FASTEX, and potentially include other parameters, although further research and evaluation is needed to determine what these parameters might be. Laboratory biomarker levels/thresholds may be considered, although there is potential for some variation between laboratories [78]; however, this variation can be overcome by considering the trend of biomarkers in addition to their absolute value, similar to the estimated glomerular filtration rate (eGFR) slope in Fabry nephropathy.

### Cardiac monitoring for treatment decisions in FD

A key need in FD is a cardiac biomarker or clinical tool that can guide treatment decision-making, specifically when to start treatment and when to switch from one treatment to another. Disease progression to chronic kidney disease in patients with FD is monitored using the decline in the eGFR slope [79]. However, a similar parameter is not yet available in cardiology.

The 2015 European Fabry Working Group Recommendations for ERT initiation considered parameters for renal function, cardiac involvement signs, gastrointestinal symptoms, and neurological symptoms [6]. The cardiac signs for the initiation of ERT were cardiac hypertrophy (MWT > 12 mm) without fibrosis, or with minimal signs of fibrosis, cardiac rhythm disturbances (sinus bradycardia, AF, repolarization disorders), and were the same irrespective of sex and disease phenotype [6].

Del Franco and colleagues proposed a different approach based on their staging system (Fig. 2), which suggests initiating FD treatment in male patients with classical FD at stage 0 (pre-hypertrophic) to prevent the development of LVH [74]. Data are limited on the value of initiating treatment at this early stage in other patients with FD [56]. However, initiating ERT or chaperone therapy in later stages may not result in slowing disease progression [74], because of pathogenic mechanisms other than substrate accumulation (e.g. inflammation, mitochondrial dysfunction) [56].

**Fig. 3:**
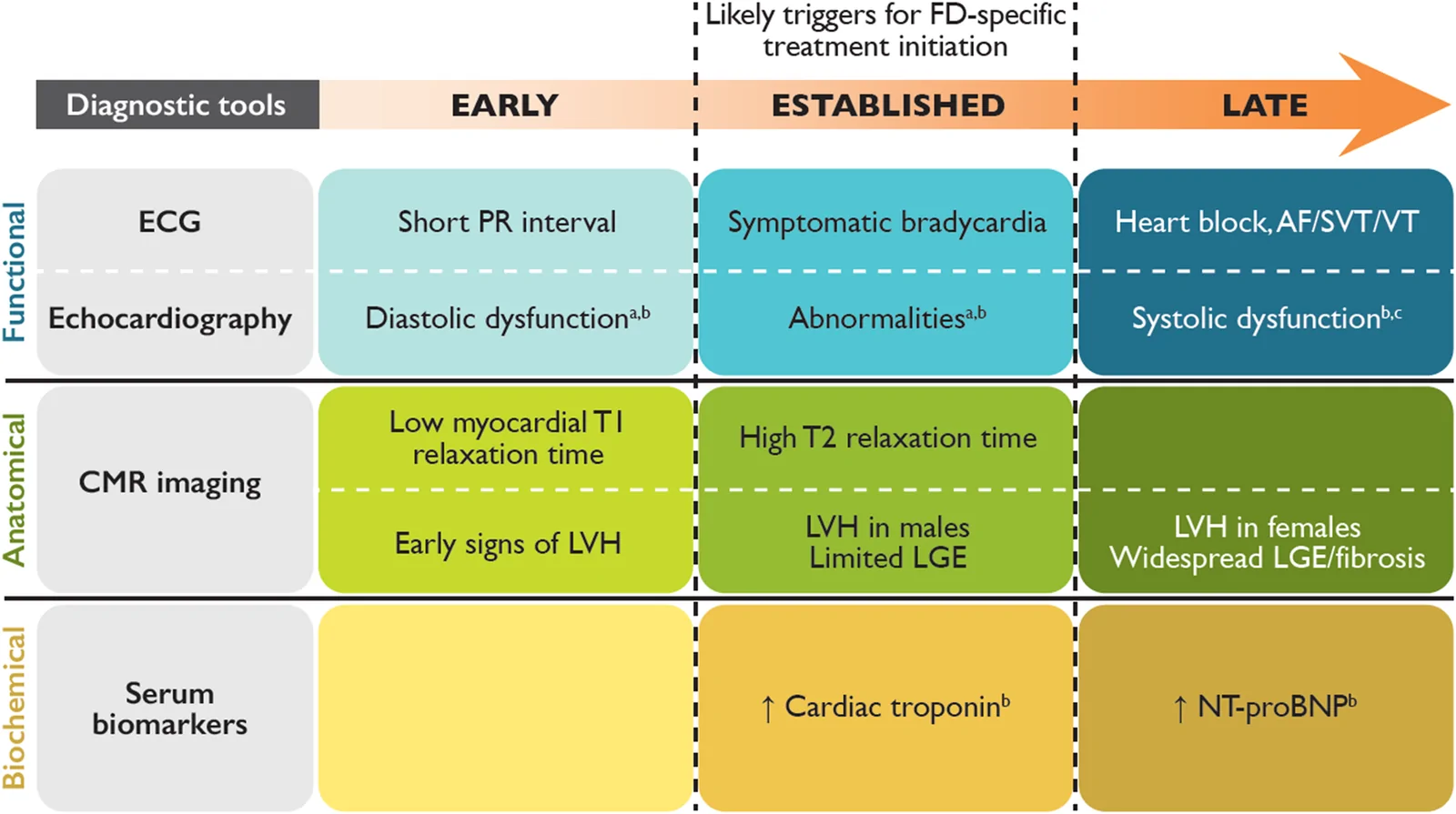
Cardiac parameters to indicate the severity of Fabry disease (FD) and triggers for treatment initiation, based on recommendations from the international PREDICT-FD consensus group [80]. ^a^Including decreased myocardial strain and strain rate; tissue Doppler abnormalities, enlarged left atrium, abnormal wall motion and pulmonary vein abnormalities; ^b^A causal relationship between this indicator and FD is needed to justify treatment initiation; ^c^Including shortened PR interval, non-supraventricular tachycardia (SVT) and symptomatic bradycardia. AF, atrial fibrillation; CMR, cardiac magnetic resonance; ECG, electrocardiogram; LGE, late gadolinium enhancement; LVH, left ventricular hypertrophy; NT-proBNP, N-terminal pro-B-type natriuretic peptide; VT, ventricular tachycardia: adapted version of Fig. 2 B from p. 8 of the article by Hughes et al. [80], originally published by and used with permission from the BMJ publishing group

An international consensus group (Proposing Early Disease Indicators for Clinical Tracking in FD or PREDICT-FD) have proposed a set of renal, cardiac, and neurological parameters that indicated the need for treatment initiation [80]. PREDICT-FD suggested important functional, anatomical and biochemical parameters of cardiac damage in FD (Supplementary Table 2) [80]. They estimated which parameters could be used to guide treatment decisions, based on whether the parameters were defined during early, established or late FD (Fig. 3). According to the PREDICT-FD consensus report, treatment should be started in all patients with established FD who display cardiac signs and in male patients of any age with classical FD and early signs of cardiac involvement, but not in female patients with early cardiac involvement [80].

While these recommendations may help guide treatment initiation, defining cardiac criteria for treatment efficacy evaluation could be more challenging. Clearance of vascular endothelial cells containing Gb3 was a surrogate endpoint in the regulatory approval of FD treatments [81]. However, this endpoint requires multiple endomyocardial biopsies (EMBs). Multiple biopsies to monitor disease progression are not feasible in daily clinical practice nor are they clearly linked to the occurrence of an outcome. Therefore, EMBs are not suitable as an endpoint in the clinical setting [82].

Echocardiographic measurements of LV wall mass are non-invasive but have limitations, specifically inherent biological, reproducibility, and interobserver variability [83]. Moreover, LV thickness is lower in women in comparison to men. In addition, the rate of LVH development must be accounted for in any staging system or endpoint evaluation [84]. For example, in the echocardiographic staging system by Meucci and colleagues, men were overrepresented in stage 1 (LVH) whereas women were overrepresented in stage 2 (left atrium dilation), possibly because a lower degree of LVH can be associated with more advanced disease in women [75].

Patient-reported outcomes are being used more frequently as primary endpoints in clinical trials. Self-reported data on cardiac parameters (palpitations or chest pain), which evaluated patient knowledge and experience in Canada and the USA provided valuable insight on symptoms, symptom frequency, and treatment with ERT and oral chaperone therapy in patients with FD [85].

### Impact of FD treatment on cardiovascular parameters

Treatments available for FD are ERTs (agalsidase-alpha or -beta, pegunigalsidase-alpha) and chaperone therapy (migalastat).

Agalsidase-beta was the first available treatment in the USA and is also available in the European Union (EU). Agalsidase-alpha is available in Europe; the pegylated form, pegunigalsidase-alpha, has been available in the USA and Europe since 2023. Data from studies using agalsidase-alpha and -beta show improvements in echocardiographic and MRI parameters of LVH [86–88], but the magnitude of the effect depends on the severity of LVH at baseline [89, 90]. In an international cohort study that retrospectively compared the effects of agalsidase-alpha (*n* = 248) and -beta (*n* = 139) on LVMI by echocardiography, patients without LVH at baseline showed no change in LVMI over 1 year, while those with LVH at baseline exhibited a decrease in LVMI in both treatment groups [89]. A higher proportion of patients in the agalsidase-beta arm (79%) had a decrease in LVMI over 1 year compared with the agalsidase-alpha arm (62%), after adjustment for baseline LVMI (odds ratio 2.27 [95% CI 1.11–4.86]; *p* = 0.03) [89].

There are limited data on the impact of pegunigalsidase-alpha on cardiovascular parameters, but the phase III BRIDGE study showed stable LVMI (measured by MRI) during 12 months of treatment in male patients (*n* = 13) [91]. LVMI increased over 12 months (versus baseline) of pegunigalsidase-alpha treatment in women (*n* = 7) with FD, but was within the normal range for age and sex [91]. Of course, due to the small sample size, the generalizability of these results should be taken with caution.

Chaperone therapy with migalastat is available in the USA and EU. In clinical trials, patients receiving migalastat had a significant reduction in LV mass on echocardiography after 12 months (*n* = 14) [92] and 24 months (*n* = 54) [93]. The changes were most marked during the first 12 months and showed little change in months 12–24 [93]. LV mass and septal T1 values were used as endpoints in the MAIORA trial [94]. In this study (*n* = 16) no significant difference was found in LVMI versus baseline after 18 months of migalastat, but it showed an increase in T2 values in the inferolateral wall (*p* = 0.02) and a small (marginally significant; *p* = 0.06) increase in septal T1 values [94]. However, due to the varied pattern of T1 values over time in FD (i.e. decrease, normalization, increase), T2 values (which consistently increase) may be a more useful parameter for assessing response to treatment.

Further research is needed to evaluate CMR parameters as endpoints in FD studies, as the lack of CRM endpoints seen in the MAIORA trial may have been the result of the small sample size and relatively short follow-up duration, rather than the inherent limitations of CMR as an assessment of outcomes.

While no significant CMR changes were apparent in the MAIORA study, exercise stress testing detected significant changes in VO_2_ at the anaerobic threshold, as well as a marginally significant change in percent predicted VO_2_ [94]. Therefore, exercise stress testing may be considered as a measure of cardiovascular treatment response.

### Unmet needs and future directions

Overall, there is no single validated cardiac parameter for elucidating the effect of FD treatment that is analogous to the eGFR slope used to gauge renal function. Rather, what is needed is a multiparametric system that includes a range of measurements and can be used for staging and treatment decision-making. This system should be simple – using readily available cardiovascular assessment techniques (e.g. ECG, laboratory biomarkers such as troponin, standard imaging modalities such as echocardiography, and CMR) – and accurate.

A number of new developments may help to improve the definition of cardiac disease progression, including more sophisticated imaging techniques such as 3D echocardiography [52], and ^18^F-fluorodeoxyglucose positron emission tomography (not easily applicable due to availability and costs) [95, 96]; implantable loop recorders to evaluate arrhythmia burden [97, 98]; and machine learning/artificial intelligence to interpret patterns in cardiac parameters [99].

**Fig. 4:**
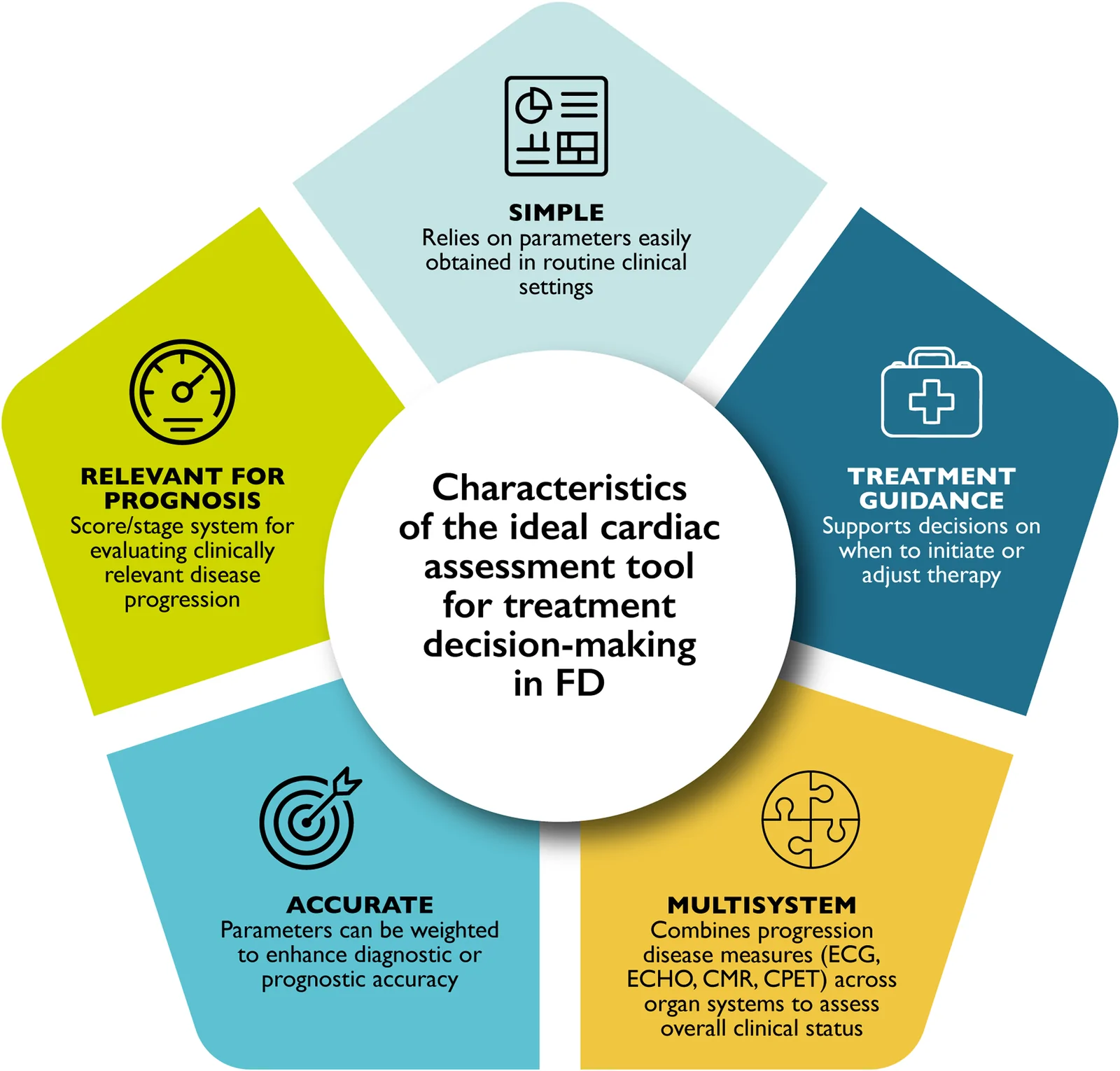
Characteristics of the ideal cardiac assessment tool for treatment decision-making in Fabry disease (FD). CMR, cardiac magnetic resonance; CPET, cardiopulmonary exercise test; ECG, electrocardiogram; ECHO, echocardiography

Cardiac assessment tools should use parameters that are easily measurable in clinical practice to aid in treatment decision-making (Fig. 4). The development of such a cardiac assessment system requires data collection from a number of centers and should include data from treatment-naïve and treated patients. Consideration should be given to the inclusion of patient-reported outcome measures, presence of arrhythmias (on ECG or loop recorder), and development of fibrosis.

**Fig. 5:**
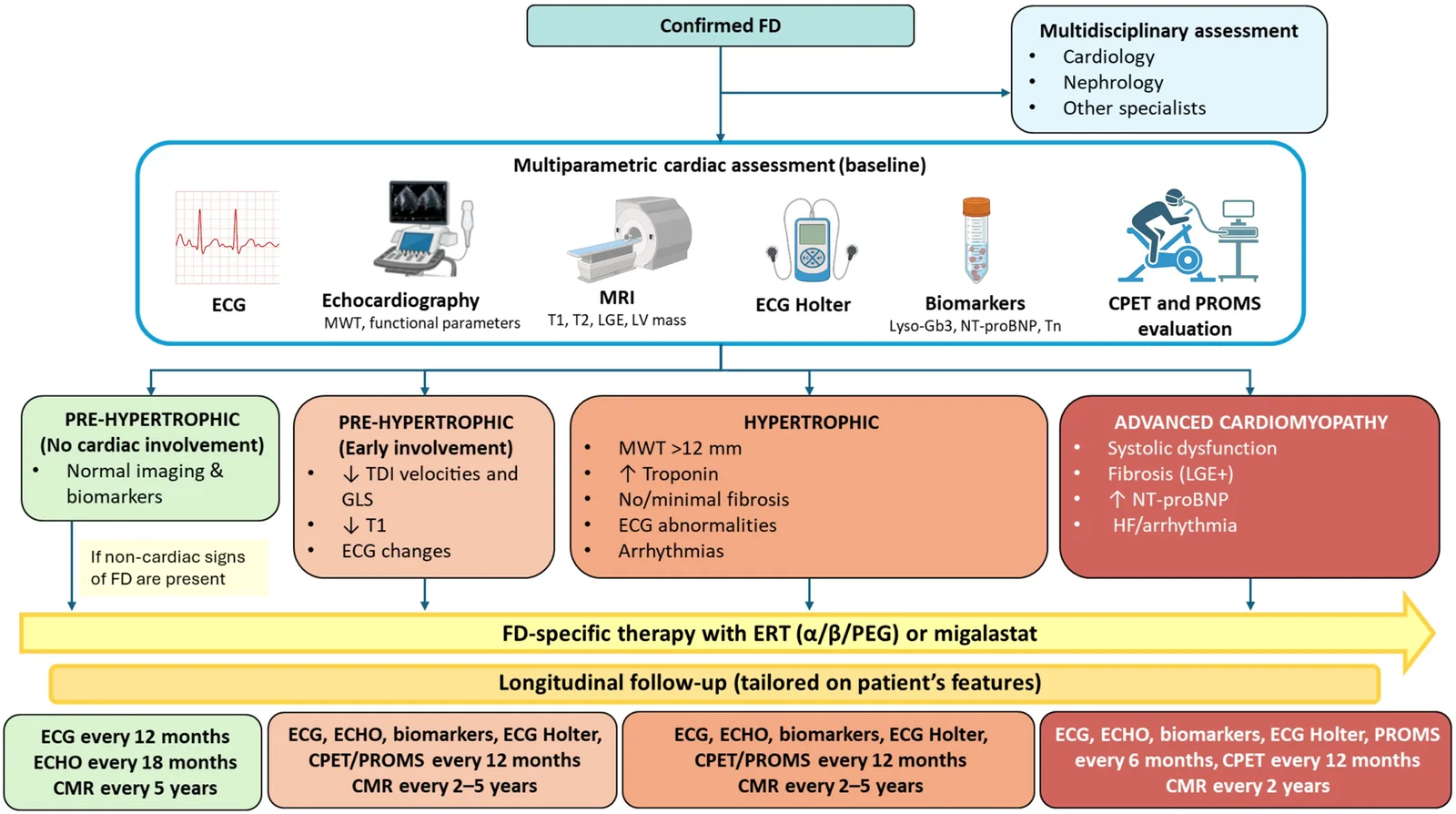
Proposed multiparametric clinical pathway for cardiac assessment, treatment initiation, and follow‑up in Fabry disease (FD). α/β/PEG, agalsidase-alpha/-beta, pegunigalsidase-alpha; CMR, cardiac magnetic resonance; CPET, cardiopulmonary exercise test; ECG, electrocardiogram; ECHO, echocardiography; ERT, enzyme replacement therapy; GLS, global longitudinal strain; HF, heart failure; LGE, late gadolinium enhancement; LV, left ventricle; LVH, left ventricular hypertrophy; lyso-Gb3, globotriaosylsphingosine; MRI, magnetic resonance imaging; MWT, maximal wall thickness; NT-proBNP, N-terminal pro-B-type natriuretic peptide; PROMs, patient-reported outcome measures; TDI, tissue doppler imaging; tn, troponin. Created with BioRender

To date, the lack of robust data on individual cardiac parameters supports the rationale for a multiparametric approach, in which the combination of imaging, functional, and biochemical parameters may overcome the limitations of any single marker. We propose a pathway (Fig. 5) that integrates cardiac assessment parameters with the stage of cardiac involvement to guide disease‑specific therapy and monitoring of response over time. It should be noted that the proposed algorithm is intended as a general guidance framework, and clinicians are expected to tailor clinical management to the individual patient’s specific characteristics and disease profile. In particular, the definition of the timing of diagnostic assessments may represent a rough approximation, as it cannot fully capture the complexity and heterogeneity inherent in each individual case. Personalized medicine remains the gold standard in patient care, and this principle is especially critical in the context of rare diseases such as FD, where phenotypic variability, organ involvement, and treatment response can differ substantially between individuals.

We intentionally excluded proposing specific parameter weightings because existing tools such as FASTEX already incorporate weighted cardiac parameters; notably, these tools have not yet been validated prospectively as a treatment response monitor. Proposing a new scoring model without prospective multicenter data would risk premature adoption of an unvalidated tool. Nevertheless, our efforts set out to define the requirements of such a tool and to advocate for the research needed to build and validate it.

Finally, since FD is a multisystem disease, effective management of patients with FD requires a multidisciplinary team. At each clinical encounter, the cardiologist should share their assessment of the patient’s cardiovascular status with other physicians involved in patient care, particularly the nephrologist (given the important relationship between renal function and cardiovascular status), so that multidisciplinary assessments are facilitated [90]. This is particularly important in FD because stability of one organ system may be accompanied by the deterioration of another [100].

## Conclusions

Although cardiovascular disease is a leading cause of death in patients with FD, an agreed staging system for evaluating cardiac progression is not available yet. We recommend the expansion of the cardiac assessment tool developed in this study to aid in treatment decision-making. Further research is needed to optimize such a system, better defining parameters and criteria, and to evaluate its prognostic and predictive validity, before it can be implemented in clinical practice.

## Electronic supplementary material

Below is the link to the electronic supplementary material.

Supplementary Material 1

## Data Availability

Data sharing is not applicable to this article as no new datasets were generated or analyzed.

## Abbreviations

α-Gal A: α-galactosidase A
AF: Atrial fibrillation
CI: Confidence interval
CMR: Cardiac magnetic resonance
E/e’: Peak early mitral inflow velocity (E) divided by the early diastolic mitral annular velocity (e’)
ECG: Electrocardiogram
EMB: Endomyocardial biopsy
ERT: Enzyme replacement therapy
FASTEV: FAbry STabilization index
FD: Fabry disease
FOS: Fabry Outcomes Survey
GLS: Global longitudinal strain
HCM: Hypertrophic cardiomyopathy
HF: Heart failure
LA: Left atrium
LAVi: Left atrial volume indexed to body surface area
LGE: Late gadolinium enhancement
LV: Left ventricular
LVEF: Left ventricular ejection fraction
LVH: Left ventricular hypertrophy
LVMI: Left ventricular mass index
lyso-Gb3: Globotriaosylsphingosine
MI: Myocardial infarction
MSSI: Mainz Severity Score Index
MWT: Maximal wall thickness
NT-proBNP: N-terminal pro-B-type natriuretic peptide
NYHA: New York Heart Association
PREDICT-FD: Proposing Early Disease Indicators for Clinical Tracking in Fabry disease
RBBB: Right bundle branch block
TAPSE: Tricuspid annual plane systolic excursion
TNF: Tumor necrosis factor
VE/VCO_2_: Ventilation/carbon dioxide production
VO_2max_: Maximum oxygen consumption
