# Supplementary material for: Cardiological aspects of Fabry disease: from diagnosis to therapeutic efficacy assessment
Source: Orphanet J Rare Dis. 2026 Jul 9;21:344. doi: 10.1186/s13023-026-04485-1 (PMC13637155; doi:10.1186/s13023-026-04485-1)
Supplement: Supplementary file 1 [file 13023_2026_4485_MOESM1_ESM.docx]

**Cardiological aspects of Fabry disease: from diagnosis to therapeutic efficacy assessment**

**Authors:** Francesca Graziani, Elena Biagini, Alessia Argirò, Rodolfo Citro, Guido Iaccarino, Chiara Lanzillo, Ines Monte, Federico Pieruzzi, Cinzia Zuchi, Maurizio Pieroni

**Corresponding author:** Francesca Graziani, MD, Department of Cardiovascular Medicine, Fondazione Policlinico Universitario A. Gemelli IRCCS, Largo A. Gemelli 8, 00168, Rome, Italy. ORCiD ID: 0000-0002-4520-5689. Telephone number: +390630154187; Email address: francesca.graziani@policlinicogemelli.it

# Supplemental materials

# Supplemental tables

## Supplemental Table 1.

Electrocardiogram (ECG) parameters that may be indicative of Fabry disease

Increased heart rate [1]

Sokolow-Lyon index [2, 3]

Left bundle branch block [3]

Right bundle branch block [4-6]

High P wave/PR segment ratio [3]

P wave morphology in the precordial lead V3 (single upright less common vs control, single inverted or biphasic leading negative more common vs control) [1]

Higher amplitude of the positive component of the P wave in lead DI [1]

Increased P wave area in the precordial lead V1 [1]

Increased P wave duration [4, 6]

Shorter PR interval [5, 6]

PQ interval ≤40 msec [6]

Increased QRS duration [3, 5, 7]

Increased QRS complex amplitude in lead I [7]

Increased time normalised QRS spatial velocity at 4/8 of the total QRS duration [1]

QRS fragmentation [4]

Higher total QRS score [5]

QT prolongation [3]

Greater QT dispersion (difference between the shortest and longest QT interval in the 12-lead ECG) [1]

R wave of ≥1.1 mV in the aVL lead [5]

Increased R wave amplitude in V5 lead [5]

Longer duration of the S wave in the precordial lead V1 [1]

Higher amplitude of the ST segment in the precordial lead V2 at 60 msec after the J point [1]

Inferior ST segment depression [5]

High T wave amplitude [3]

Giant T waves (positive in early disease and negative in later disease) [4]

## Supplemental Table S2.

The international PREDICT-FD group’s current and/or future early indicators of cardiac damage for use in routine clinical practice, by order of importance [8]. Future parameters are those not currently available across all centres

| **Order** | **Parameter** |
| --- | --- |
| **Current** | |
| 1= | Early indicators of LVH |
| 1= | Late Gd enhancement on CMR imaging |
| 3= | Reduced myocardial T1 relaxation time of CMR imaging |
| 3= | Elevated serum cardiac troponin |
| 3= | Abnormal ECG |
| 3= | Abnormal echocardiogram |
| 3= | Early indicators of histological damage (heart biopsy) |
| 8 | Markers of early systolic/diastolic dysfunction |
| 9 | Elevated serum NT-proBNP |
| 10 | Abnormal wall motion |
| **Current or future** | |
| 1= | Reduced myocardial T1 relaxation time on CMR imaging |
| 1= | Elevated serum cardiac troponin |
| 3 | Elevated serum NT-proBNP |

CMR, cardiac magnetic resonance; ECG, electrocardiogram; Gd, gadolinium; LVH, left ventricular hypertrophy; NT-proBNP, N-terminal pro-B-type natriuretic peptide

# References

1.	Namdar M, Richardot P, Johner N, Shah D, Nordbeck P, Olivotto I, et al. Recognition of pre-hypertrophic cardiac involvement in Fabry Disease based on automated electrocardiographic measures. Int J Cardiol. 2021;338:121-126.

2.	Camporeale A, Pieroni M, Pieruzzi F, Lusardi P, Pica S, Spada M, et al. Predictors of clinical evolution in prehypertrophic Fabry disease. Circ Cardiovasc Imaging. 2019;12:e008424.

3.	Figliozzi S, Camporeale A, Boveri S, Pieruzzi F, Pieroni M, Lusardi P, et al. ECG-based score estimates the probability to detect Fabry disease cardiac involvement. Int J Cardiol. 2021;339:110-117.

4.	Parisi V, Baldassarre R, Ferrara V, Ditaranto R, Barlocco F, Lillo R, et al. Electrocardiogram analysis in Anderson-Fabry disease: a valuable tool for progressive phenotypic expression tracking. Front Cardiovasc Med. 2023;10:1184361.

5.	Vitale G, Ditaranto R, Graziani F, Tanini I, Camporeale A, Lillo R, et al. Standard ECG for differential diagnosis between Anderson-Fabry disease and hypertrophic cardiomyopathy. Heart. 2022;108:54-60.

6.	Antezana-Chavez E, Cianciulli TF, Hadid CL, Toro DD, Celano L, Saccheri MC, et al. Value of electrocardiography to distinguish Fabry disease from sarcomeric hypertrophic cardiomyopathy. Am J Cardiol. 2022;178:131-136.

7.	Zada M, Lo Q, Trivedi SJ, Harapoz M, Boyd AC, Devine K, et al. Electrocardiographic characteristics and their correlation with echocardiographic alterations in Fabry disease. J Cardiovasc Dev Dis. 2022;9:11.

8.	Hughes DA, Aguiar P, Deegan PB, Ezgu F, Frustaci A, Lidove O, et al. Early indicators of disease progression in Fabry disease that may indicate the need for disease-specific treatment initiation: findings from the opinion-based PREDICT-FD modified Delphi consensus initiative. BMJ Open. 2020;10:e035182.
